# Visible-light active conducting polymer nanostructures with superior photocatalytic activity

**DOI:** 10.1038/srep18002

**Published:** 2015-12-11

**Authors:** Srabanti Ghosh, Natalie Amoin Kouame, Samy Remita, Laurence Ramos, Fabrice Goubard, Pierre-Henri Aubert, Alexandre Dazzi, Ariane Deniset-Besseau, Hynd Remita

**Affiliations:** 1 Laboratoire de Chimie Physique, UMR 8000-CNRS, Bât. 349, Université Paris-Sud, Université Paris-Saclay, 91405 Orsay, France; 2 Département CASER, Ecole SITI, Conservatoire National des Arts et Métiers, CNAM, 75141 Paris Cedex 03, France; 3 Laboratoire Charles Coulomb (L2C), UMR 5221 CNRS-Université de Montpellier, Montpellier, France; 4 Laboratoire de Physicochimie des Polymères et des Interfaces (LPPI), Université de Cergy-Pontoise, 95031 Cergy-Pontoise Cedex, France; 5 CNRS, Laboratoire de Chimie Physique, UMR 8000, 91405 Orsay, France

## Abstract

The development of visible-light responsive photocatalysts would permit more efficient use of solar energy, and thus would bring sustainable solutions to many environmental issues. Conductive polymers appear as a new class of very active photocatalysts under visible light. Among them poly(3,4-ethylenedioxythiophene) (PEDOT) is one of the most promising conjugated polymer with a wide range of applications. PEDOT nanostructures synthesized in soft templates *via* chemical oxidative polymerization demonstrate unprecedented photocatalytic activities for water treatment without the assistance of sacrificial reagents or noble metal co-catalysts and turn out to be better than TiO_2_ as benchmark catalyst. The PEDOT nanostructures exhibit a narrow band gap (E = 1.69 eV) and are characterized by excellent ability to absorb light in visible and near infrared region. The novel PEDOT-based photocatalysts are very stable with cycling and can be reused without appreciable loss of activity. Interestingly, hollow micrometric vesicular structures of PEDOT are not effective photocatalysts as compared to nanometric spindles suggesting size and shape dependent photocatalytic properties. The visible-light active photocatalytic properties of the polymer nanostructures present promising applications in solar light harvesting and broader fields.

The development of efficient and environmentally friendly approaches for energy conversion and storage is one of the pivotal challenges of the 21^st^ century^1^. Nanomaterials potentially provide paradigm changing solutions to energy conversion processes that occur at surfaces^2^,^3^. Titanium dioxide (TiO_2_) is currently the most widely used photocatalyst because of its high photocatalytic activity, its stability, low cost and non-toxicity. Nevertheless, TiO_2_ applications are limited because of a low quantum yield with the fast charge carriers (e^−^/h^+^) recombination and the necessity to use UV irradiation. Indeed, TiO_2_ can only be excited by UV irradiation with wavelengths shorter than 400 nm and it absorbs only 3-4% of the solar light impinging on the Earth’s surface^4^,^5^,^6^. In the last few years, a considerable number of novel strategies including doping, heterojunctions, graphene-based composition and co-catalyst have been proposed to offer new photocatalytic materials as potential substitutes of TiO_2_ for the most relevant photocatalytic applications such as detoxification and disinfection, water splitting and organic synthesis^7^,^8^,^9^. To achieve high visible light-induced photocatalytic activity, it is crucial to finely tune several electronic characteristics of photocatalysts such as atomic configuration, bandgap energy, band position, lifetime of electrons and holes, etc.^9^,^10^. Several approaches, including the use of dopants such as nitrogen or carbon have been used to narrow the band gap of TiO_2_^4^. Recently, black hydrogenated TiO_2_ nanocrystals with high photocatalytic activity under solar light have been developed^11^. Another example, plasmonic photocatalysts have appeared as a very promising way to enhance the activity of TiO_2_ in the visible light^12^,^13^. Moreover, surface modification of TiO_2_ with Cu, Ag or Au nanoparticles significantly enhance the photocatalytic activity under UV and visible light^14^,^15^,^16^. However, the high cost of the noble metal doped catalyst (i.e., Au, Ag) and the low stability with repeated cycling significantly limit their large-scale applications^17^. Hence, the existing photocatalysts have not yet found way for practical applications.

In the last few years, polymer nanostructures have attracted a lot of attention for energy conversion and storage applications^18^. In this field, we have very recently reported the first experimental evidence of a visible light responsive photocatalytic activity of conjugated poly(diacetylene)-based polymers nanostructures, poly(diphenylbutadiyne) (PDPB) nanofibers for water depollution and have shown the crucial role of the material structure at a nanometric scale^19^. Poly(3,4-ethylenedioxythiophene) (PEDOT) on the other hand is one of the most promising conducting polymers because of its excellent thermal and chemical stability, high conductivity, flexibility, low-cost, high transparency and elevated carrier mobility as well as biocompatibility^20^. These unique characteristics render PEDOT potentially useful for a wide range of applications, including solar cells, organic light emitting devices, and biosensors^21^,^22^,^23^. Although oxide-based semiconductors have been in vogue as efficient photocatalysts in the past decade, the reports regarding photocatalytic activity of conjugated polymers are scarce^18^,^24^. In the present work, we show that conducting PEDOT polymer nanostructures exhibit exceptionally high and shape dependent-photocatalytic activities both under UV and visible light, which is higher than that of plasmonic Ag-TiO_2_ and of poly(diphenylbutadiyne) (PDPB) nanofibers recently published^18^. We present here the first illustration of employing pure PEDOT as a very efficient photocatalyst for the depollution of water.

We have developed a soft template mediated controlled synthesis of PEDOT nanostructures with tunable morphology. Hexagonal mesophases composed of oil-swollen surfactant-stabilized tubes arranged on a triangular lattice in salted water and doped with EDOT monomers in the oil phase were used as soft templates (Fig. 1a)^25^. Polymer nanostructures are produced *via* chemical oxidative polymerization of EDOT monomers using FeCl_3_ as an oxidizing agent as shown in Fig. 1b 26. The polymerization occurs by stepwise RC-RC (radical cation, RC) coupling of oxidized EDOT monomers or oligomers in the presence of an oxidant (Supplementary Scheme S1^27^. The hexagonal mesophases are perfectly transparent, stable gels and after polymerization turned into a translucent dark blue gel (Inset: Fig. 1c i,ii, Supplementary Fig. S1). The pure dark blue solid powder of PEDOT was extracted from the mesophases (by addition of ethanol) and used for photocatalytic activity evaluation. Depending on the mesophase composition, spindle-like or vesicle-like PEDOT structures are obtained as shown in Fig. 1d,e. The PEDOT spindle nanostructures are 40 nm thick and several hundred nanometers long, while the PEDOT vesicles are spherical hollow capsules of diameter around 1 μm with walls of thickness around 40 nm (Supplementary Fig. S2). Figure 1f,g illustrates an AFM topographic image and the corresponding 3D AFM image of well dispersed PEDOT spindle nanostructures with a regular shape. In contrast, aggregated network polymer structures (denoted as bulk PEDOT) were obtained in bulk solution (without using surfactant) confirming the templating effect of the mesophases (Supplementary Figure S3). Our methodology offers simple processability under ambient conditions which is pre-requisite for industrial applications of conducting polymers. Figure 1c displays the UV-visible absorption spectrum of the extracted polymer nanostructures showing a peak at 390 nm along with a broad absorption band in the near IR-region related to polaron/bipolaron subgap transitions. In contrast to vesicle or spindles shaped PEDOT, bulk PEDOT demonstrates only a slight absorbance in the visible region (Supplementary Fig. S4).

Waste waters generated by the textile industries contain considerable amounts of non fixed dyes and especially of azo-dyes which are toxic to aquatic life and create serious environmental pollution. Methyl orange (an azo dye) is a fairly stable and persistent dye pollutant under visible light irradiation without a photocatalyst. The photocatalytic activities of PEDOT were evaluated for the degradation of phenol and methyl orange (MO) (taken as model pollutants) in water under UV and visible light. The blank test showed that almost no photolysis of MO was observed under similar experimental conditions. At the same time, the dark absorption test (phenol and MO with initial concentration of 3.7 × 10^−3^ mol L^−1^ and 6.1 × 10^−5^ mol L^−1^ respectively) in the presence of the polymer (vesicles, nanospindles and bulk) was studied prior to the photocatalytic tests but showed no significant change in the concentration of phenol and MO in solution (Supplementary Fig. S5a–c). To compare the photocatalytic activities of commercial TiO_2_ (P25, from Evonik, known to be one of the best active photocatalysts under UV light) and of silver modified P25 TiO_2_ (Ag-TiO_2,_ active under visible irradiation)^14^ with that of PEDOT nanospindles, PEDOT vesicles and bulk PEDOT (1 mg/mL), a series of photodegradation experiments were carried out using phenol and MO under both UV and visible light. Figure 2a displayed the degradation of phenol by the different photocatalysts under UV-visible light and show clearly that the PEDOT nanospindles exhibit the highest catalytic activity. A complete degradation of phenol has been obtained for TiO_2_, Ag-TiO_2_, and PEDOT nanospindles, after irradiation for 60 min, 15 min, and 10 min respectively. However, only 30% degradation has been achieved for PEDOT vesicles under identical reaction conditions. In fact, poly(diphenylbutadiyne) (PDPB) nanofibers exhibited a UV light induced photocatalytic activity with 82% degradation of phenol after long irradiation (270 min) but this activity is even lower than that of bare or modified TiO_2_^19^. Importantly, the PEDOT nanospindles showed a high photocatalytic activity under visible light: 100% of phenol was degraded after 240 min irradiation (wavelengths > 450 nm), compared to less than 20% for the plasmonic photocatalyst Ag-P25 for the similar irradiation time (Fig. 2b). In contrast, bulk PEDOT showed a weak photocatalytic activity under both UV and visible light (Supplementary Fig. S6).

For the degradation of MO under UV light, similar degradation efficiency is displayed in Fig. 2(c). The PEDOT nanospindles displayed a significant MO photodegradation achieving 100% degradation after 180 min irradiation under visible light (Fig. 2d). Interestingly, PEDOT nanospindles showed an efficient photocatalytic activity under UV light both for phenol and methyl orange and the photocatalytic activity was much higher than that of P25 TiO_2_ (Fig. 2a,c), which is recognized as one of the best photocatalyst under UV light. Notably, PEDOT vesicles remained totally inactive for both phenol and MO degradation under visible light. For the degradation of MO under UV-visible light, similar degradation efficiency of PEDOT nanospindles has been displayed in Fig. 2c. Remarkably, 100% degradation of MO was achieved by using PEDOT nanospindles after 15 min UV light irradiation. The PEDOT spindles displayed a significant MO photodegradation achieving 100% degradation after 180 min under Visible light irradiation (Fig. 2d). It has to be noted that the photocatalytic activity of PEDOT nanospindles have been found to be even higher than the one recently reported for PDPB nanofibers^19^ under UV and visible light for phenol and MO degradation (Fig. 2): 100% of phenol is degraded with PEDOT nanospindles after 240 min irradiation under visible light, while only 64% of phenol is degraded with PDPB.

One of the key features of a photocatalyst is that it must be efficiently recycled and reused after repeating cycles of degradation reactions. Therefore, a set of experiments has been conducted to study the stability of the photocatalysts. The photocatalytic activity of PEDOT spindles was retained at over 98% and 95% of its original activity for phenol and MO respectively after six successive experimental runs as shown in Fig. 3a. This suggests that PEDOT nanospindles could be efficiently recycled and reused for repeating cycles without appreciable loss of activity, which promoted the PEDOT photocatalyst for its practical applications in the field of environmental protection. The total mineralization of the organic pollutants has been followed using a common technique, the disappearance of the total organic carbon (TOC) for expressing the detoxification level of water. In addition to a prompt removal of the colors, polymer-based photocatalysis was simultaneously able to fully oxidize the organic pollutant and dye, with an almost complete mineralization of carbon into CO_2_ and H_2_O (Fig. 3b). TOC measurements indicate ∼90% of mineralization of phenol after 240 min under both UV and visible light. These results suggest that the conducting polymer nanostructures are able to mineralize organic pollutants. It has to be noted that with PDPB nanofibers, only ∼50% mineralization was achieved after 270 min irradiation under visible light in the same irradiation conditions^19^.

The polymer structures were also characterized by the combination of a nanoscale probe from an atomic force microscope with tunable infrared (IR) source using a nanoIR instrument that can survey the various regions of the polymer via AFM topography imaging^25^,^28^. The morphology and chemical structure of the PEDOT nanospindles remained unchanged after different cycles of photocatalytic degradation reactions (Fig. 3c–e). The peak**s** around 1360 and 1476 cm^−1^, due to C-C and C = C stretching of the quinoïdal structure of the thiophene ring, indicated the effective presence of PEDOT. After five successive experimental runs of photodegradation of phenol, the band obtained at 1476 cm^−1^ which originates from the C = C stretching of the quinoïdal structure of the thiophene ring in the PEDOT polymer did not change and no new peak was observed in the PEDOT nanospindles, which indicates a high stability of the polymer nanostructures. Hence, the conjugated polymer nanostructures, because of their excellent photocatalytic activity and stability, constitute a new generation of photocatalysts active not only under UV-irradiation but also under visible-light for environmental remediation.

Photocatalysis involves generation of charge carriers, i.e. excess electrons (e^−^) and holes (h^+^), and the catalytic reactions induced by these species. A high photocatalytic activity is closely related to an efficient separation of the photoexcited electron-hole pairs generated in the photocatalyst after its excitation. The organic pollutants and dyes can be photodegraded *via* a photocatalytic oxidation process^7^,^11^. A large number of major reactive species including holes, HO^•^ and O_2_^•−^ radicals are involved in the photocatalytic oxidation process^18^. Therefore, the effects of scavengers on the degradation of phenol were examined in attempt to elucidate the reaction mechanism. As a consequence of quenching, photocatalytic oxidation reactions may be partly suppressed, and the catalytic efficiency lowered. The higher photocatalytic efficiency is reduced by scavengers, the more important the role the corresponding oxidizing species plays in the photocatalytic oxidation reaction. The effects of a series of scavengers on the degradation efficiency of phenol are shown in Supplementary Fig.S7. Photodegradation tests under argon atmosphere have been performed to address the crucial role of oxygen. In this case, the photodegradation efficiency of PEDOT nanospindles for phenol degradation under UV and visible light irradiation was reduced to 40% and 22%, respectively. These results obtained under deaerated conditions suggest a suppression of O_2_^•−^ radical (E^0^(O_2_/O_2_^•−^) = −0.33 V_SHE_)^29^ production during degradation of the model pollutant by PEDOT.


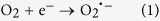


To probe the role of the excess electrons in the photocatalytic process, experiments were conducted in the presence of Cu^2+^ as scavenger of electrons. Addition of Cu^2+^ induces a decrease in the photodegradation efficiencies to 48% and 28%, respectively corroborating the role of excess electrons in the photocatalytic process. The excess electrons react with Cu^2+^ to yield Cu^+^. This reaction, which is in competition with reaction (1), causes a decrease in the production of O_2_^•−^ in the photocatalytic system (Supplementary Fig. S7), which in turn slows down the degradation kinetics. To determine the contribution of HO^•^ formed on the surface of the PEDOT photocatalysts under visible light illumination, Hantzsch method was used via the quantification of formaldehyde using Tris(hydroxymethyl)aminomethane (Tris) as a probe^30^. During the reaction between Tris and hydrogen abstracting species such as hydroxyl radical, formaldehyde is produced which can correlate the formation of HO^•^ generated on the surface of the PEDOT during photocatalysis reaction. It can be seen that formation of HO^•^ increases in the photocatalytic oxidation process with irradiation time as shown in Supplementary Fig. S8 both under UV and visible light irradiation.

In order to understand the mechanistic aspects of visible light responsive photocatalysis, cyclic voltammetry (CV) measurements were used to calculate the experimental HOMO and LUMO energy levels from the ionization potential and the electronic affinity, respectively and determine the band gap of PEDOT nanospindles (Fig. 4a). We found that the main *p-*doping (oxidation) and *n-*doping (reduction) are irreversible processes, and the values of the peak potentials *vs.* Ag/AgCl are + 0.139 V (oxidation) and −1.552 V (reduction) for PEDOT nanospindles. This reveals the onset of oxidation and reduction processes occurring at lower potentials having a much lower energy gap around 1.69 eV than TiO_2_ (3.2 eV). When illuminated with photons of energy exceeding (or equal to) the band gap (E ≥ 1.69 eV or λ ≤ 733 nm), excess electrons and holes are formed in the conjugated polymer chains.

Based on the band gap structure of the as-prepared polymer nanostructures and the effects of scavengers, a possible photocatalysis mechanism for degradation of organic pollutants and dyes can be proposed. Due to low band gap of the PEDOT, less energy is required to promote an electron to the conduction band and as a result more electronic transitions are likely to occur at higher wavelength radiation (less energetic) compared to TiO_2_ or even PDPB. Upon excitation and promotion of an electron from the valence band to the conduction band (across the band-gap) of a semiconducting material, there exists a tremendous electrostatic driving force to recombine electrons and holes which becomes a major rate-limiting factor in a catalytic process. Based on the analyses, the proposed schematic diagram of photoexcited electron-hole separation process is shown in Fig. 4b. Under visible-light irradiation, a photon (with energy higher that the band gap) excites an electron from the valence band (VB) to the conduction band (CB) of PEDOT nanospindles. Hence the electrons can easily migrate to the surface of the PEDOT and react with oxygen to form oxidizing O_2_^•−^ superoxide radical. This effective separation of photogenerated electron–hole thereby promotes superior photocatalytic activity. Meanwhile, the photogenerated holes on PEDOT (E_CB_, + 0.139 vs Ag corresponding to + 0.667 eV vs. SHE) cannot produce hydroxyl radicals. These species have a very high oxidation potential (E^0^(HO^•^/ H_2_O) = 2.27 V_SHE_) and are involved in the photooxidation reactions with TiO_2_. However, HO^•^ radicals can be formed by the following reactions (Equation 2, 3, 4, 5):


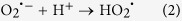



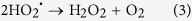



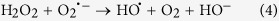



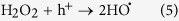


The results show that the photocatalysis mechanism involves O_2_^•−^, photo induced h^+^ , and HO^•^ radicals mediated degradation of organic pollutant with effective charge separation in PEDOT nanospindles (Fig. 4).

Hence, the uniform PEDOT nanospindles with high visible light absorption compared to its bulk counterpart (bulk PEDOT does not absorb significantly visible light, Supplementary Fig. S4), could account for the enhanced photocatalytic activity and improved stability which is consistent with PDPB nanofibers (Supplementary Fig. S6a,b). On the other hand, the absorption spectra of both nanospindles and vesicle polymer structures have similar profiles (not shown here), but the conductivity of PEDOT vesicles (7 × 10^−2^ S/cm) is much lower in comparison to that of PEDOT nanospindles (0.4 S/cm). Although both polymer structures can absorb light in visible region, photo-induced electrons are less mobile (i.e. lower conductivity) in PEDOT vesicle structures and the recombination of charge carriers may be faster. Hence less electrons could escape the recombination and reach the surface. This probably explains the lower photocatalytic degradation of organic molecules observed for PEDOT vesicles compared to PEDOT nanospindles. Both PEDOT structures (vesicles and nanospindles) showed no X-ray diffraction pattern indicating that they were not well crystallized. The difference in the photocatalytic activity between nanospindles, vesicles and bulk PEDOT might be due to larger size and to the presence of more defects in vesicles (sub-micron size) and bulk PEDOT favoring higher e^−^–h^+^ recombination. Note that such a behavior has also been recently observed in the case of PDPB, where the photocatalytic activity was much higher for nanostructures than for the bulk polymer (Supplementary Fig. S6a,b). These results show the importance of the nanostructuration and morphology of the conjugated polymer in photocatalytic activity. This dependence of the photocatalytic activity on the size and morphology has also been observed in the case of semiconductors such as TiO_2_^31^,^32^. The application of conjugated nanostructures in the field of photocatalysis can be generalized to other polymers.

In summary, Poly(3,4-ethylenedioxythiophene) nanostructures have been synthesized by a facile and reproducible chemical oxidative method using hexagonal mesophases as soft templates at room temperature. The PEDOT nanostructures with much narrower band gap (E = 1.69 eV) compared to TiO_2_ (or even to PDPB nanostructures) have therefore excellent ability to absorb light in visible and near infrared regions. Our results demonstrate that PEDOT nanostructures show exceptional high photocatalytic activities under UV (even higher than P25-TiO_2_) and visible light (higher than PDPB nanofibers) and are of huge potential for environmental remediation. This photocatalytic activity appears only at the nanoscale range. We show that the photocatalytic activity depends also on the shape of the PEDOT nanostructures. The PEDOT nanospindles have been found to be very stable photocatalysts with cycling. The photocatalytic oxidation reactions involve the oxidative O_2_^•−^ and HO^•^ species. Our results demonstrate that conducting polymer nanostructures constitute a new class of photocatalysts for environment remediation. Other applications of these nanostructures in self-cleaning surfaces, and hydrogen generation are under investigation.

## Methods

### Reagents

3,4-ethylenedioxythiophene (EDOT), iron (III) chloride, cetyltrimethylammonium bromide (CTAB) (≥98%), sodium chloride, toluene (>99%), pentanol (≥99%), silver perchlorate AgClO_4_ (>98%), phenol (C_6_H_5_OH) and methyl orange were purchased from Sigma-Aldrich. Titania (P25, surface area = 50 m^2^ g^_1^, 80% Anatase, 20% Rutile) was obtained from Evonik for comparative photo degradation by polymer nanostructure. N_2_ gas (purity > 99.995%) was purchased from Air Liquide. All compounds were used as received. Ultrapure water (Millipore System, 18.2 MΩ cm) and ethanol (≥99% for HPLC, purchased from Sigma-Aldrich) were used as solvents. All experiments were performed at room temperature.

### Synthesis of Polymer in Mesophases

The swollen hexagonal mesophases were prepared following the previously published method with some modifications. This system exhibits oil in water (O/W) direct phase structure made of hexagonally packed nonpolar cylinders filled by toluene and stabilized by a monolayer of cationic surfactants and cosurfactants, which are surrounded by a continuous water domain. Additionally, the swelling of the mesophases was varied by changing the volume ratio of oil over water (O/W). We varied both O/W ratio and NaCl concentration simultaneously. For example, mesophases with O/W = 1.5 and NaCl at 0.1 M or with O/W = 2.5 and NaCl at 0.3 M were prepared at fixed EDOT concentrations. The hexagonal mesophases were made of a mixture of cetyltrimethylammonium bromide (CTAB) as surfactant, salted water (NaCl), toluene as oil and pentanol as cosurfactant. Typically, 1.03 g of CTAB was dissolved in 2 mL of an aqueous solution containing salt (0.1 M NaCl). After a vigorous agitation for few minutes at 50 °C, the surfactant was completely dissolved giving a transparent and viscous micellar solution. The subsequent addition of 2.98 mL of toluene in the micellar solution under stirring induced the formation of a white unstable emulsion. The cosurfactant (20 μL of pentanol) was then added to the mixture which was strongly vortexed for a few minutes. This led to a perfectly translucent, birefringent and stable gel consisting in a hexagonal mesophase for PEDOT vesicles structures. For another composition (with O/W = 2.5 and NaCl at 0.3 M), 4.42 mL of toluene and 30 μL of pentanol have been used to prepare the mesophases following the same methodology for PEDOT nanospindles. According to the same procedure, other mesophases were also prepared in the presence of both the monomer (EDOT) and the oxidant (FeCl_3_). EDOT and/or FeCl_3_ were dissolved separately in toluene and added to the viscous micellar solution during mesophases preparation. Evidently, EDOT and FeCl_3_ were never mixed together before this step in order to avoid bulk polymerization.

The swollen hexagonal mesophases were also prepared for the synthesis of PDPB polymer nanostructure. Typically, 1 g of the surfactant (Sodium Dodecyl Sulfate) was dissolved in 2 mL of 0.3 mol.L^−1^ NaCl in glass tubes. After a vigorous agitation at 30 °C, the surfactant had completely dissolved to give a transparent and viscous micellar solution. The subsequent addition of cyclohexane containing monomer 1,4-diphenylbutadiyne (DPB) (10% of mass) and initiator benzoin methyl ether (BME) (1%) in the micellar solution under stirring leads to a white unstable emulsion. A cosurfactant, pentanol-1 (420 μL), was then added to the mixture, which was then strongly vortexed for a few minutes. This led to a perfectly colorless, translucent, birefringent and stable gel: a hexagonal mesophase. The doped mesophases with the monomer and the initiator for polymerization were used as soft templates to synthesize polymer nanostructures induced by irradiation using UV light with an Oriel 300 W Xenon UV-visible lamp at a distance of 5 cm for 12 hours.

### Extraction of the polymer nanostructures

After reaction, the materials were extracted in a water-ethanol mixture, centrifuged, and washed several times to eliminate the surfactant, the cosurfactant and the salts.

### Synthesis of silver modified TiO_2_

The photocatalysts were obtained by radiolytic reduction of Ag^+^ in the TiO_2_ suspension (2 wt %). An ethanolic solution containing AgClO_4_ (2 × 10^−3^ M) and TiO_2_ (P25) in suspension is first sonicated for 3 min, degassed with nitrogen, and irradiated (under stirring) with a ^60^Co panoramic gamma source (dose rate = 2.3 kGy h^−1^). The silver ions were reduced by the solvated electrons and the alcohol radicals induced by solvent radiolysis and 1 h 20 min exposure time (3.2 kGy) was necessary to reduce all the silver ions. The modified TiO_2_ photocatalysts were separated by centrifugation and dried at 60 °C. The modified catalyst (Ag-TiO_2_) has been used for methyl orange and phenol degradation.

### Photocatalytic Activity Measurements

The photocatalytic activity under UV-visible illumination of the PEDOT has been tested by photodecomposition of phenol (C_6_H_5_OH) in water. The light beam emitted from Oriel 300 W xenon lamp was passed through an IR water filter and a UV cut-off filter (λ > 450 nm) before being focused onto a cylindrical Pyrex reactor through a quartz window. The photodegradation reaction of phenol was carried out using a 10 mm optical path quartz cell reactor containing 3.5 mL of 3.7 × 10^−3^ mol L^−1^ of phenol in the presence of 1 g L^−1^ of bulk, vesicle or spindles PEDOT. The PEDOT was added in the phenol solution and magnetically stirred for 10 minutes in the dark to ensure the adsorption equilibrium of phenol and MO prior to irradiation. The solution was then irradiated with a xenon lamp (under UV and visible light) and bubbled with O_2_ at a fixed flow rate along with magnetic stirring. A 0.5 mL aliquot was collected from the reactor at different time points. In case of P25, after degradation reaction, the solution was centrifuged to separate the catalyst and obtain a transparent solution. High-performance liquid chromatography (HPLC) was used to determine the concentration of phenol and to study its degradation. A Varian Prostar 230 ternary gradient pump was combined with a Prostar 330 photodiode array detector (D2 lamp). For elution, an isocratic mobile phase consisting of 75% H_2_O and 25% acetonitrile (ACN), at a 1 mL min^−1^ flow rate, was used, with detection at 270 nm. The column was an Adsorbosphere C18 reverse phase (5 μm, *l*: 150 mm, ID: 4.6 mm, Alltech) combined with an All-Guard cartridge system^TM^ (7.5×4.6 mm, Alltech). For data acquisition, Star software was used.

For the photodegradation of methyl orange (MO), the conditions were the same as those used for phenol except that the initial concentration of MO was 6 × 10^−5^ mol L^−1^. MO solutions were characterized using a HP Agilent diode array 8453 UV-visible spectrophotometer and following the signal at the wavelength of 464 nm, which corresponds to the maximal absorption.

Multiple photocatalysis experiments were performed under identical reaction conditions to determine reproducibility. After the completion of the degradation of MO and Phenol, PEDOT spindles were recovered by filtration, and then washed thoroughly with water. The recovered PEDOT spindles were dried at 30 °C overnight. This used catalyst was re-employed in the next cycle under identical conditions.

### Hantzsch method for the determination of formaldehyde

In this method, 1.5 mL of reactant solution was extracted after photo irradiation of organic pollutants in the presence of PEDOT spindles, TiO_2_ (P_25_TiO_2_), Ag nanoparticle modified TiO_2_ (Ag-TiO_2_) diluted in 1 mL of 0.2 M acetoacetanilide in ethanol and 2.5 mL of 4 M ammonium acetate. Consequently, a dihydropyridine has been formed having a maximum absorption wavelength of 368 nm. The absorbance has been measured by a spectrophotometer.

Experiments with the total organic carbon (TOC) technique were conducted to study the mineralization of phenol and MO at different irradiation times. TOC was measured by using a Shimadzu TOC-LCSH. TOC was measured by IR after complete oxidation by catalytic combustion at 680 °C on exclusive platinum catalyst, the inorganic carbon being removed by a previous acidification and air purging.

## Additional Information

**How to cite this article**: Ghosh, S. *et al.* Visible-light active conducting polymer nanostructures with superior photocatalytic activity. *Sci. Rep.* **5**, 18002; doi: 10.1038/srep18002 (2015).

## Supplementary Material

Supplementary Information

## Figures and Tables

**Figure 1:**
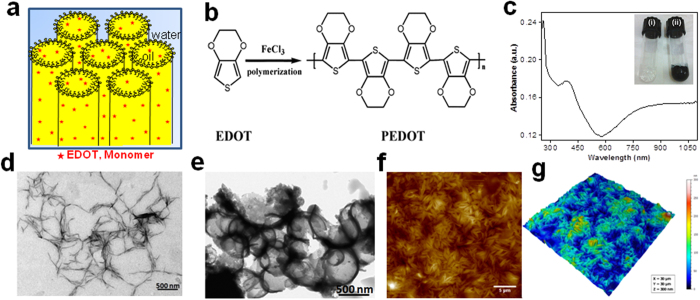
Synthesis and characterization of PEDOT polymer. (**a**) Hexagonal mesophases doped with 3,4-ethylenedioxythiophene (EDOT) monomer. (**b**) Schematic representation of chemical oxidative polymerization of 3,4-ethylenedioxythiophene (EDOT) using FeCl_3_ as chemical oxidant. **(c)** Absorption spectrum of ethanolic solution of PEDOT nanospindles. Inset: Photograph of doped swollen hexagonal phases synthesized in presence of 0.3 M NaCl. (i) Transparent gel before polymerization and (ii) dark blue gel after polymerization by FeCl_3_. (**d**) Transmission electron micrograph (TEM) of PEDOT nanospindles synthesized in presence of 0.3 M NaCl. (**e**) TEM image of PEDOT vesicles synthesized in presence of 0.1 M NaCl. (**f**) Typical AFM topographic image of PEDOT nanospindles extracted from mesophases and deposited onto ZnSe substrate. (**g**) The corresponding 3D AFM image of PEDOT nanostructures.

**Figure 2:**
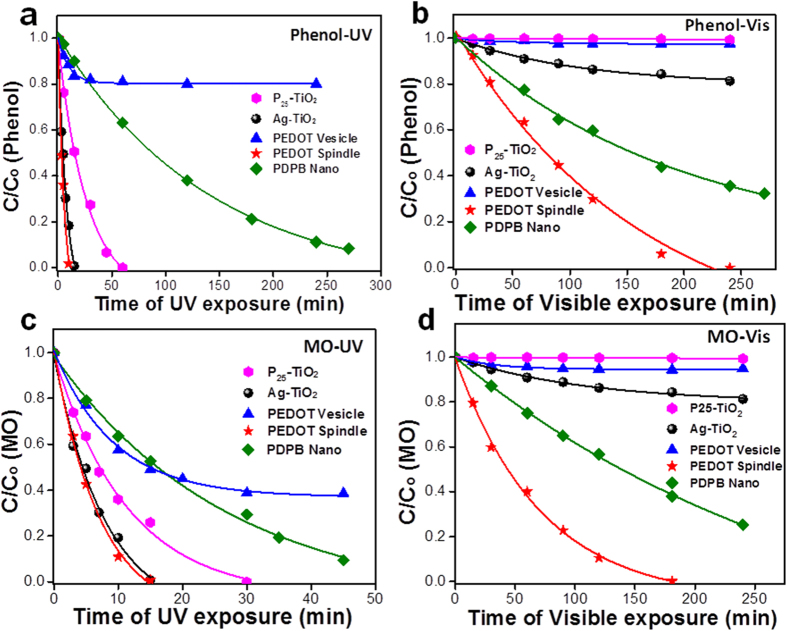
Comparative photocatalytic activities of polymer vesicles, nanospindles, TiO_2_ and Ag-TiO_2_. Photocatalytic degradation of (**a,b**) phenol and (**c,d**) methyl orange (MO) in the presence of commercial P25 TiO_2_ and Ag-TiO_2_, PDPB nanofibers and the synthesized PEDOT vesicles and PEDOT nanospindles under UV (**a,c**) and visible light (>450 nm) (**b,d**) irradiation. The concentrations of PDPB nanofibers, PEDOT vesicles, PEDOT nanospindles, Ag-TiO_2_ and TiO_2_ in water were 1 mg/mL. Initial concentrations C_0_ were 3.7 × 10^−3^ mol L^−1^ for phenol and 6 × 10^−5^ mol L^−1^ for MO.

**Figure 3:**
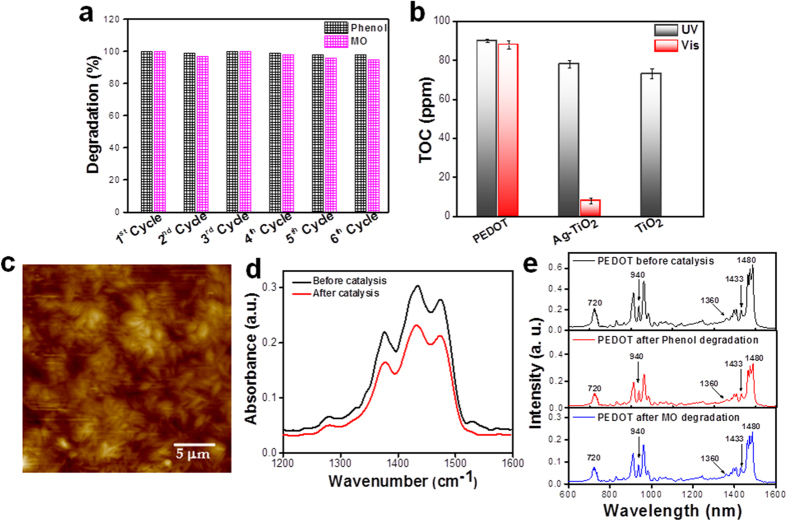
Recycling and stability of the PEDOT nanospindles. (**a**) Recyclability of the PEDOT nanospindles photocatalysts in six successive experiments for the photocatalytic degradation of Phenol and MO in aqueous solution under visible light irradiation. (**b**) Comparative data of total organic carbon (TOC) disappearance of phenol at natural pH after 240 min irradiation under visible light. (**c**) Topographic image of PEDOT nanospindles by conventional AFM after degradation of phenol. (**d**) NanoIR spectra recorded spectral regions of the PEDOT polymer before and after 6 cycles catalysis. (**e**) The attenuated total reflectance (ATR)-Fourier transformed infrared (FTIR) spectra of PEDOT nanostructures before (top black spectrum) and after six cycles photocatalytic degradation of phenol (middle red spectrum) and methyl orange (bottom blue spectrum) under visible light irradiation.

**Figure 4:**
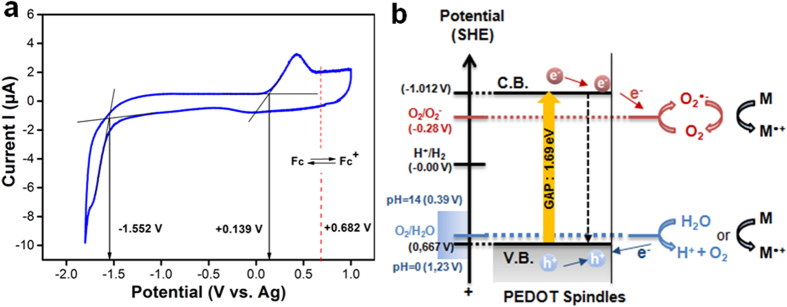
The photocatalytic mechanism and determination of energy level of polymer structures. (**a**) Cyclic voltammograms of PEDOT nanospindles obtained at 20 mV/s in acetonitrile, 0.1 M Tetrabutylammonium Perchlorate. Ferrocenium/ferrocene (Fc/Fc^+^) redox potential has been measured at the end of the experiment in order to calibrate the pseudo reference electrode (0.63 V *vs*. Ag in the present study). The energetic levels of PEDOT nanospindles is determined as follows: E_HOMO_ (eV) ~ ionization potential = −4.8− e (E_ox_onset_ −0.63) and E_LUMO_ (eV) ~ electronic affinity = −4.8−e (E_red_onset_ −0.63). (**b**) Photocatalysis mechanism with charge separation in PEDOT nanospindles, with electron reducing oxygen and hole oxidizing water.
