# Supplementary material for: Visible-light active conducting polymer nanostructures with superior photocatalytic activity
Source: Sci Rep. 2015 Dec 11;5:18002. doi: 10.1038/srep18002 (PMC4676008; doi:10.1038/srep18002)
Supplement: Supplementary Information [file srep18002-s1.pdf]

Supplementary Information

Visible-light active conducting polymer nanostructures with superior photocatalytic activity

Srabanti Ghosh[1], Natalie Amoin Kouame[1], Samy Remita[1, 2], Laurence Ramos[3], Fabrice Goubard[4], Pierre-Henri Aubert[4], Alexandre Dazzi,[1] Ariane Deniset-Besseau,[1] and Hynd Remita*[,1,5]

[1]Laboratoire de Chimie Physique, UMR 8000-CNRS, Bât. 349, Université Paris-Sud, Université Paris Saclay, 91405 Orsay, France
[2]Département CASER, Ecole SITI, Conservatoire National des Arts et Métiers, CNAM, 75141 Paris Cedex 03, France
[3] Laboratoire Charles Coulomb (L2C), UMR 5221 CNRS-Université de Montpellier, Montpellier, France.
[4]Laboratoire de Physicochimie des Polymères et des Interfaces (LPPI), Université de Cergy-Pontoise, 95031 Cergy-Pontoise Cedex, France
[5]CNRS, Laboratoire de Chimie Physique, UMR 8000, 91405 Orsay, France
*corresponding author, E-mail: hynd.remita@u-psud.fr

Overview.

1. Characterization

2. Scheme S1

3. Figure S1-S8

Material Characterization.

The morphology eventually combined with local spectrum of synthesized PEDOT on solid substrate was determined by combining the classical atomic force microscope (AFM) with tunable pulsed laser as the IR source. We have used a commercial setup, nanoIR (Anasys Instrument corp.) allowing us to cover the range from 3600 cm^{-1} to 1000 cm^{-1}. Usually, samples are directly deposited on the upper surface of a ZnSe prism that is transparent in the mid-infrared and the tip of the AFM remains in contact with the object. When the sample absorbs the IR laser pulse, it warms via the photothermal effect, resulting in a rapid thermal expansion of the absorbing region of the sample. The thermal expansion pulse impacts the tip of the AFM cantilever and causes it to oscillate. As the amplitude of oscillations is proportional to the absorption, we are able to record infrared absorption spectra at a given point and to make chemical maps by scanning the surface at a given wavelength. In this study, drops of ethanolic solution of powder before and after catalytic degradation were directly deposited on the upper surface of a ZnSe prism and dried at room temperature. The IR source has been tuned to a 3054 cm^{-1} to map compositional variations in order to identify further change of the polymer materials at the specific location after photocatalytic degradation reaction.

The attenuated total reflectance (ATR)-Fourier transformed infrared spectroscopy (FTIR) of PEDOT spindles before and after catalytic degradation was obtained using a Brüker Vertex 70 FTIR spectrometer with diamond ATR attachment (PIKEMIRACLE crystal plated diamond/ZnSe) and MCT detector with liquid nitrogen cooling system. Scanning wavelengths were varied from 4000−600 cm^{-1} with a 4 cm^{-1} spectral resolution with 100 repetitions scans average for each spectrum.

Cyclic voltammetry (CV) was used to estimate energetic levels (E_{HOMO}, E_{LUMO}) by the determination of ionization potential (IP) and electronic affinity (EA) of an organic semiconductor. CV measurements of bulk was carried out in a glovebox using a Princeton Applied Research Potentiostat/Galvanostat model 273A (EG&G, The Netherlands). The electrochemical setup was a three-electrode single cell, with a glassy carbon (GC) disk (2 mm diameter) coated with the sample film as the working electrode, a Pt wire as counter electrode, and a silver wire pseudo reference electrode. Ferrocenium/ferrocene (Fc/Fc^{+}) redox potential was measured at the end of each experiment in order to calibrate the pseudo reference electrode as

recommended by IUPAC. Polymer samples (C = 1 mg/mL in tetrahydrofuran) were drop-casted onto the GC-electrode and then dried. The GC-electrode was immersed into the electrochemical cell containing acetonitrile with 0.1 M tetrabutylammoniumhexafluorophosphate (TBAPF6). The compounds were electrochemically reduced prior to being oxidized between −3.0 V and+3.0 V at a scan rate of 20 mV/s.

The spin-coated films were fabricated on glass slides at 1000 rpm for 60s using the PEDOT ethanolic solutions obtained after extraction from mesophases. The thickness (ca. 200-500 nm thickness) of the films was measured using a 3 Veeco Dektak 150 surface profiler. The electrical conductivity of the polymer films was measured using a Kelvin four-point probe technique implemented with Keithley 2420 system.

Material Characterization.

Small-angle X-ray scattering (SAXS) was used to characterize the pure hexagonal mesophases and the mesophases doped with the monomer and with the catalyst before and after polymerization. The mesophases were inserted in glass capillaries of 1.5 mm diameter. A high brightness low power X-ray tube, coupled with aspheric multilayer optic (GeniX 3D from Xenocs) was employed, which delivered an ultralow divergent beam (0.5 mrad). Scatterless slits were used to give a clean 0.8 mm diameter X-ray spot with an estimated flux around 35 Mph/s at the sample position. A transmission configuration was used. The scattered intensity was collected on a two-dimensional Schneider 2D image plate detector prototype, at a distance of 1.9 m from the sample. The experimental data were corrected for the background scattering and the sample transmission. The scattering vector q can be calculated from the angle of the scattered radiation and X-ray wavelength. The scattering pattern of a hexagonal phase consists in diffraction peaks whose positions are in the ratio 1:√3:2. The first peak position q_0 allows a direct determination of the hexagonal lattice parameter a according to $a = \frac{2}{\sqrt{3}}\frac{2\pi}{q_0}$.

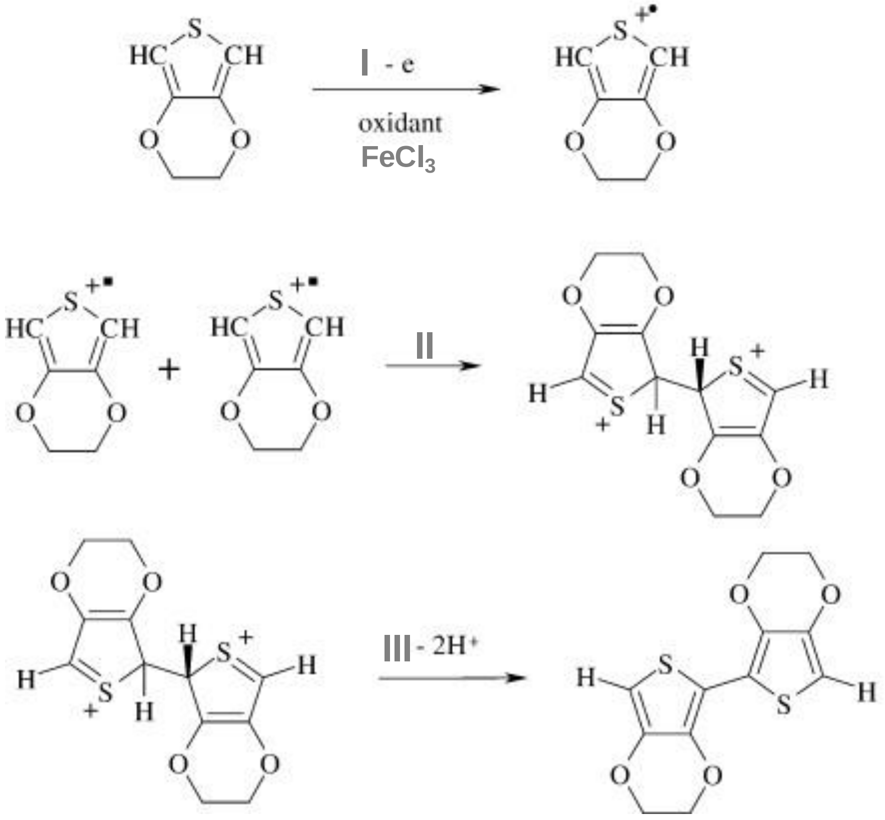

Scheme S1 Chemical oxidative polymerization of EDOT monomer using $FeCl_3$ as chemical oxidant.

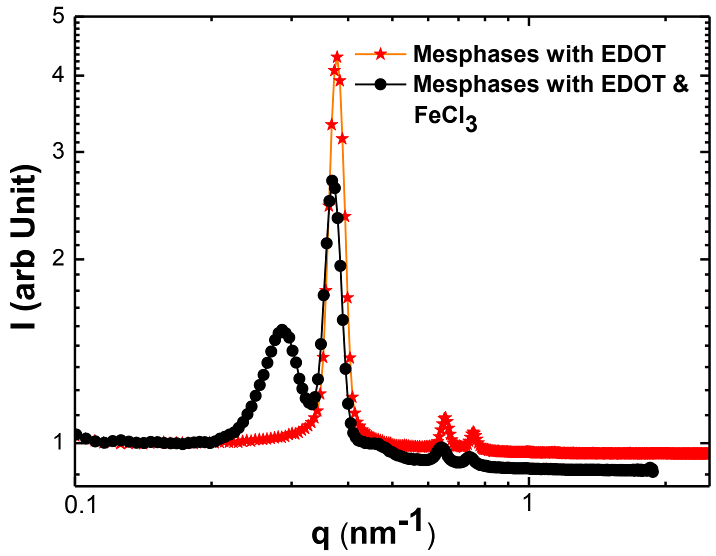

Figure S1 Small-angle X-ray scattering spectra of monomer doped swollen hexagonal phases (red stars) and after polymerization of EDOT by using $FeCl_3$ (black circles). The diffraction patterns are characteristic of a hexagonal phase as demonstrated by three Bragg peaks whose positions are in the ratio 1:√3:2. From the peak position and sample composition, one evaluates that the diameter of the oil-swollen surfactant stabilized tubes is 19.1 nm.

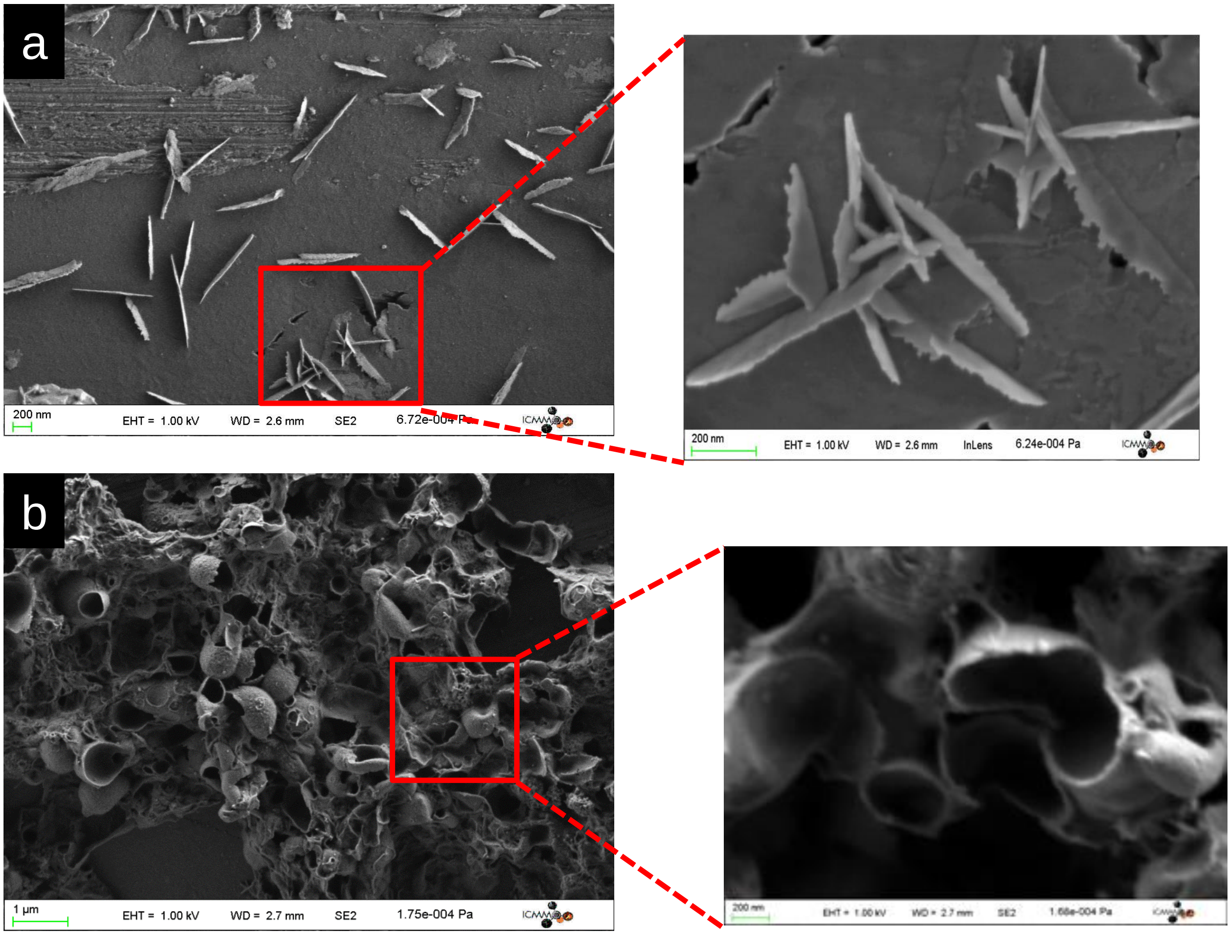

Figure S2 SEM images of PEDOT structures deposited on aluminium substrate **(a)** PEDOT vesicles (with a zoom) synthesized into mesophases at 0.1 M NaCl **(b)** spindle-shaped PEDOT (with a zoom), synthesized into mesophases at 0.3 M NaCl in the presence of 0.1 M EDOT and 0.1 M $FeCl_3$.

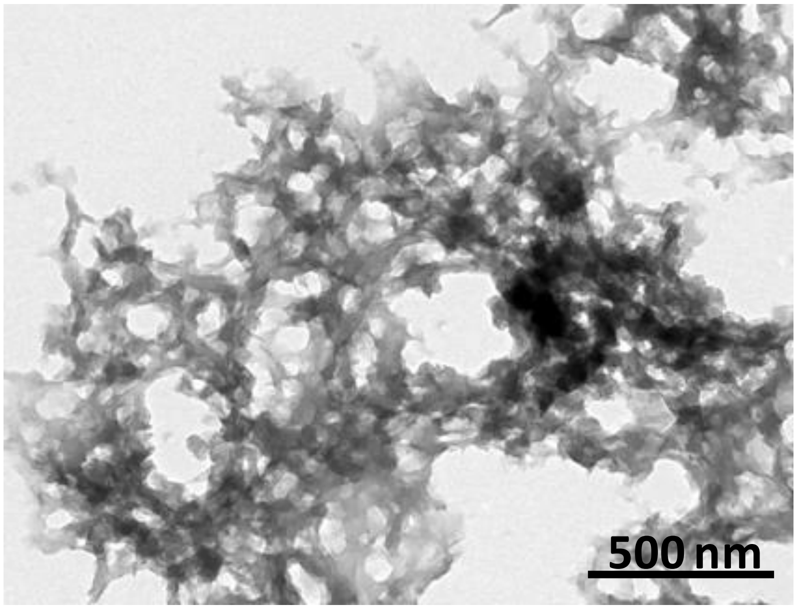

Figure S3 TEM image of PEDOT polymers synthesized in bulk solution (without using the swollen hexagonal mesophases). 0.1 M in EDOT and 0.1 M in $FeCl_3$ were used for the bulk PEDOT preparation.

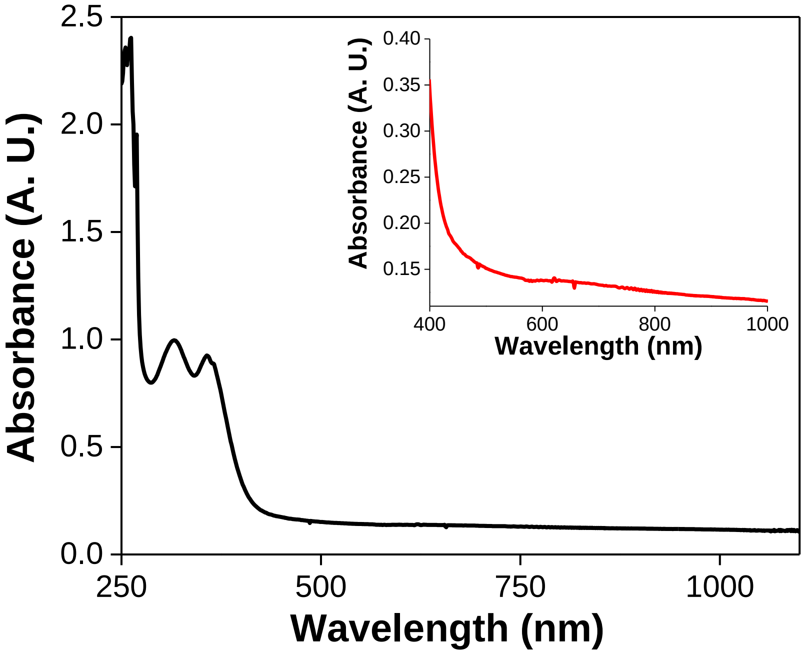

Figure S4 Absorption spectrum of ethanolic solution of bulk PEDOT using 0.1 M EDOT and 0.1 M $FeCl_3$ as chemical oxidant for the oxidation of EDOT monomers. The optical path length was 0.1 cm. The reference was ethanol. Inset: absorption spectra of bulk PEDOT monitored at 400-1000 nm.

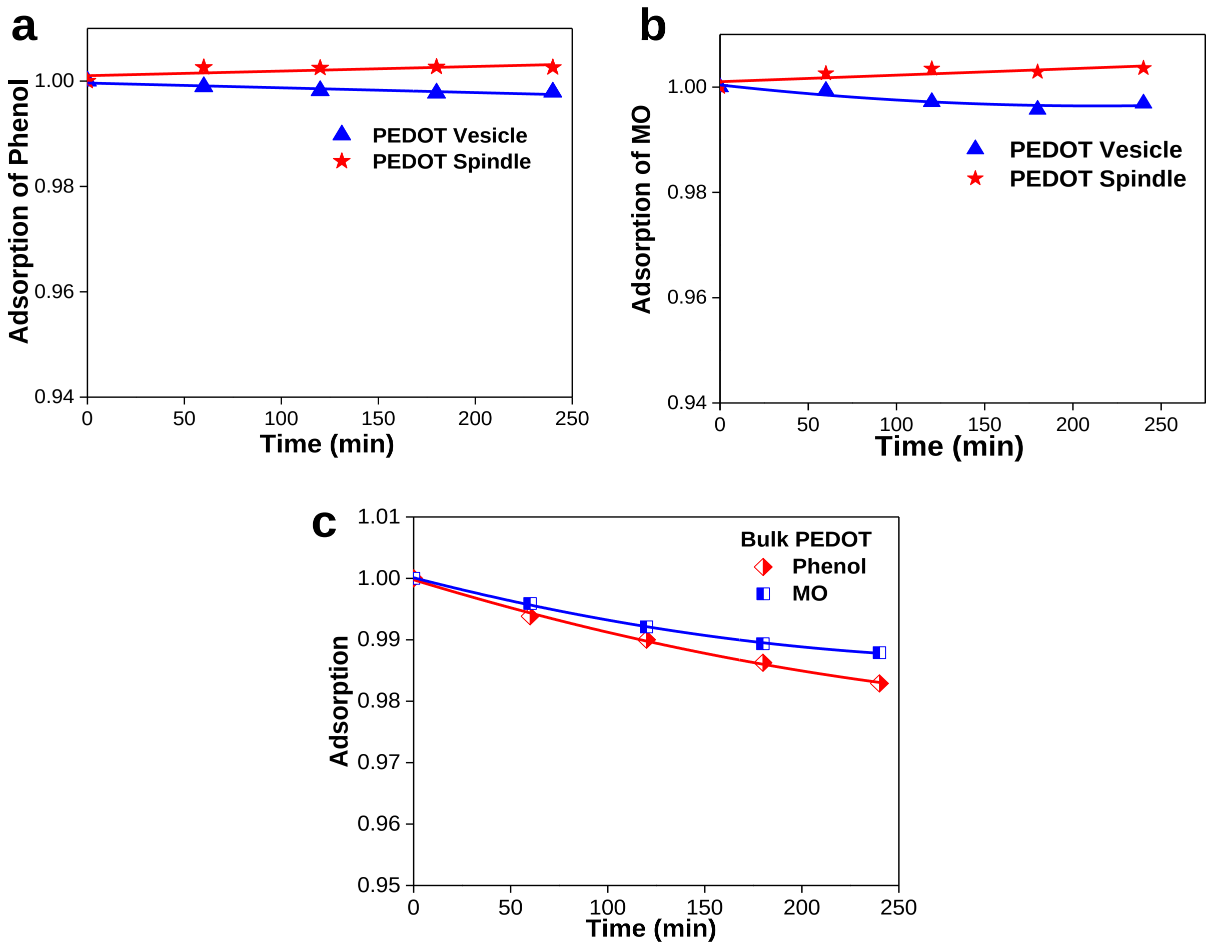

Figure S5 The dark adsorptions tests of PEDOT vesicles, PEDOT spindles and bulk PEDOT. (a) Phenol and (b) Methyl orange (MO) on PEDOT vesicles (blue triangles), PEDOT spindles (red star). (c) Adsorption of both phenol and MO on bulk PEDOT. The initial concentrations are C_0= 3.7 × 10^{-3} mol L^{-1} for phenol and C_0=6 × 10^{-5} mol L^{-1} for MO.

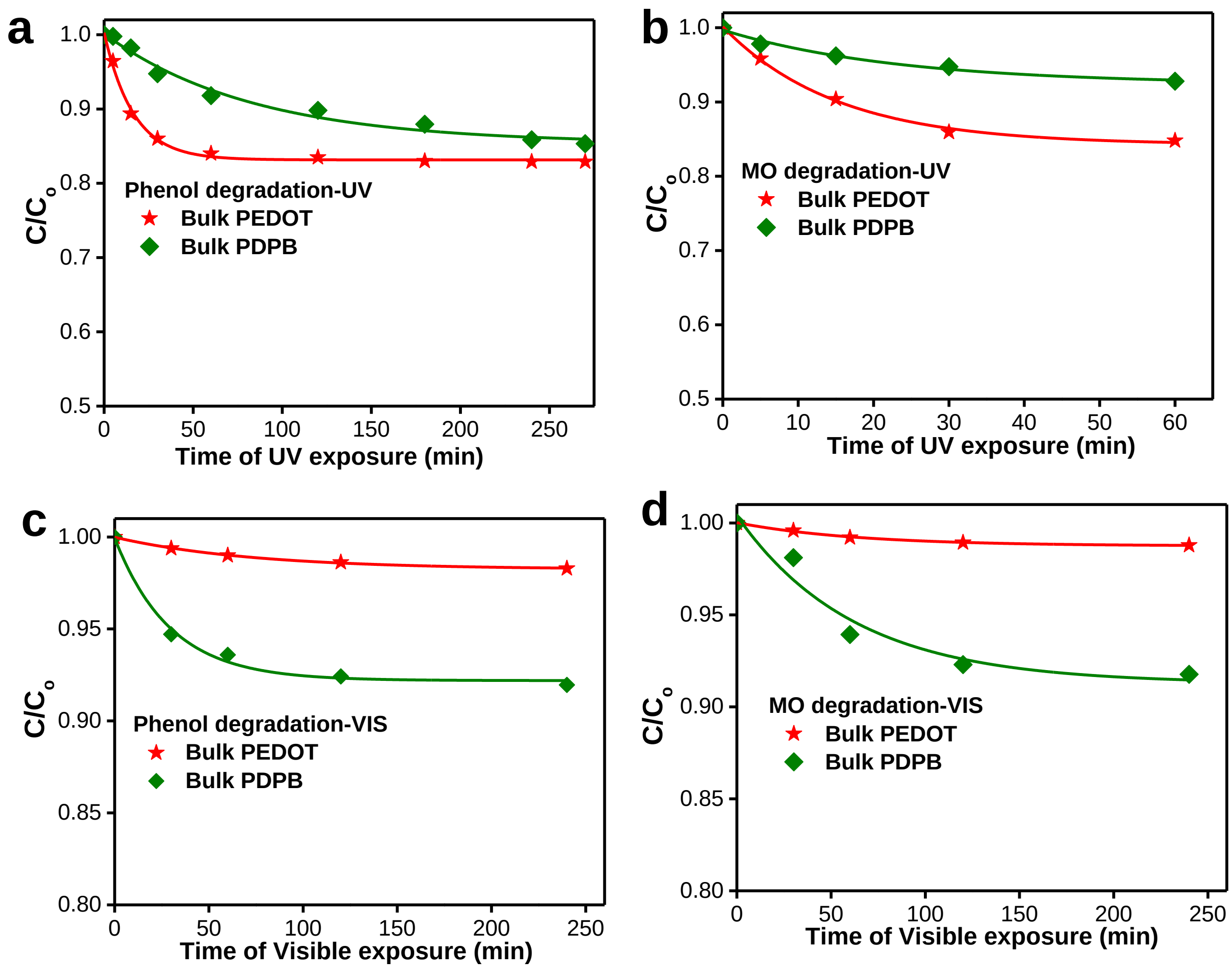

Figure S6 Comparative photocatalytic activity of bulk PEDOT. Figure shows the degradation of phenol and MO under irradiation with (a, b) UV and (c, d) visible light. The initial concentrations are $C_0 = 3.7 \times 10^{-3}$ mol L^{-1} for phenol and $C_0 = 6 \times 10^{-5}$ mol L^{-1} for MO.

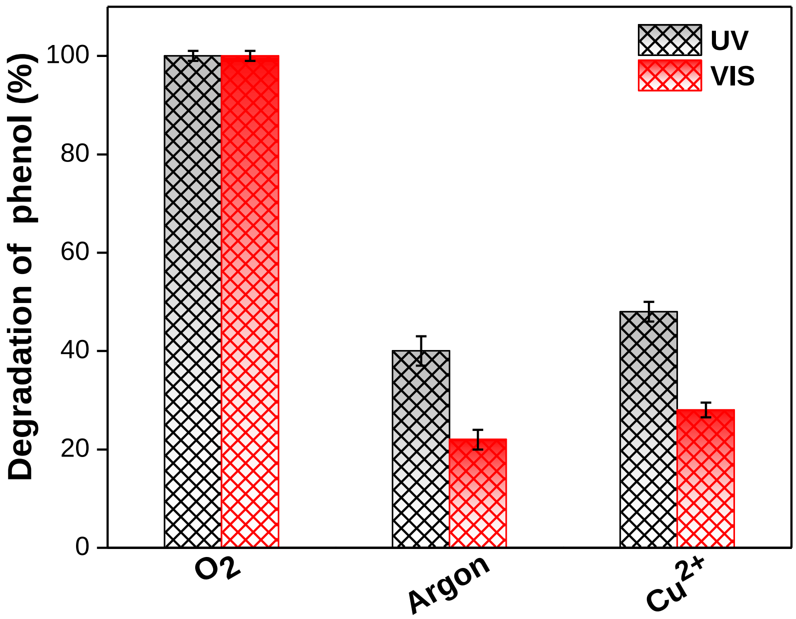

Figure S7 Effect of oxygen, argon and Cu^{2+} on the photocatalytic activity of PEDOT spindles for the degradation of phenol under UV-visible and visible light (λ> 450 nm), irradiation after 10 and 240 minutes, exposure to light respectively. (Initial concentrations C_0 for phenol is 3.7×10^{-3} mol L^{-1}).

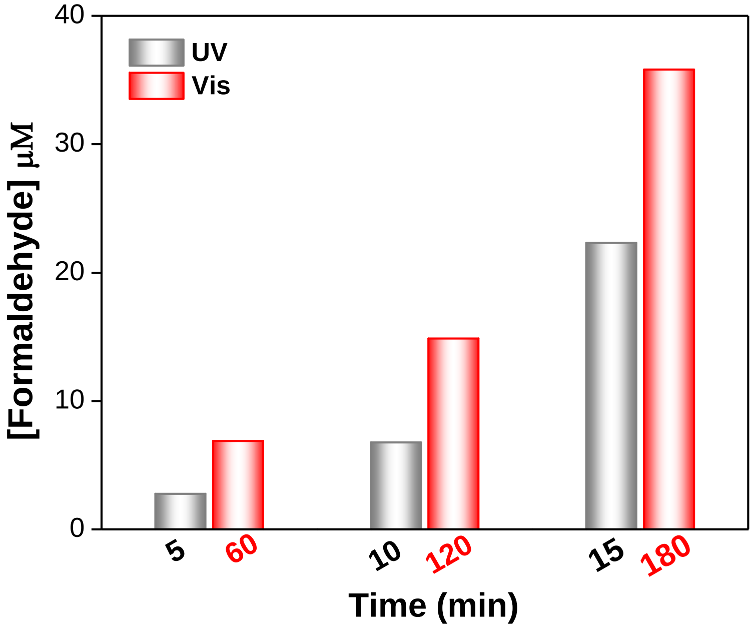

Figure S8 Determination of formaldehyde concentration using Tris as a probe for hydroxyl radical generation after photodegradation of phenol in the presence of PEDOT spindles under UV light and visible light at different time interval.
